# Targeting hypertension with SGLT2 inhibition: the EU-funded SGLT2 HYPE trial

**DOI:** 10.1093/eurheartj/ehag301

**Published:** 2026-05-15

**Authors:** Ingo Eitel, Roza Saraei, Elias Rawish

**Affiliations:** Medical Clinic II, University Heart Center Lübeck, Ratzeburger Allee 160, Lübeck 23538, Germany; German Centre for Cardiovascular Research (DZHK), Partner Site North, Lübeck, Germany; Medical Clinic II, University Heart Center Lübeck, Ratzeburger Allee 160, Lübeck 23538, Germany; German Centre for Cardiovascular Research (DZHK), Partner Site North, Lübeck, Germany; Medical Clinic II, University Heart Center Lübeck, Ratzeburger Allee 160, Lübeck 23538, Germany; German Centre for Cardiovascular Research (DZHK), Partner Site North, Lübeck, Germany

## A European investigator-initiated trial to close the evidence gap in hypertension care

Hypertension remains the leading modifiable risk factor for cardiovascular morbidity and mortality worldwide.^1^ Despite the availability of multiple antihypertensive drug classes and clear guideline recommendations, blood pressure control remains suboptimal in a substantial proportion of patients. Across Europe, more than 80 million individuals are affected, and fewer than 40% achieve recommended blood pressure targets despite treatment.^2^ Consequently, hypertension continues to be a major driver of myocardial infarction, stroke, heart failure, atrial fibrillation, chronic kidney disease, and premature death, with substantial medical and socioeconomic consequences. Current antihypertensive strategies primarily focus on blood pressure reduction. However, even in patients achieving guideline-recommended targets, a considerable residual cardiovascular risk persists. This observation highlights the need for novel therapeutic approaches that go beyond blood pressure lowering and directly target the pathophysiological consequences of hypertension.

The *SGLT2 HYPE trial (SGLT2 Inhibition for Cardiovascular Endpoint Reduction in Hypertension)* was designed to address this unmet need. It is the first large-scale, randomized, outcome-driven trial specifically evaluating whether sodium–glucose cotransporter-2 (SGLT2) inhibition improves cardiovascular and renal outcomes in patients with hypertension.

## Rationale for SGLT2 inhibition in hypertension

**Figure 1:**
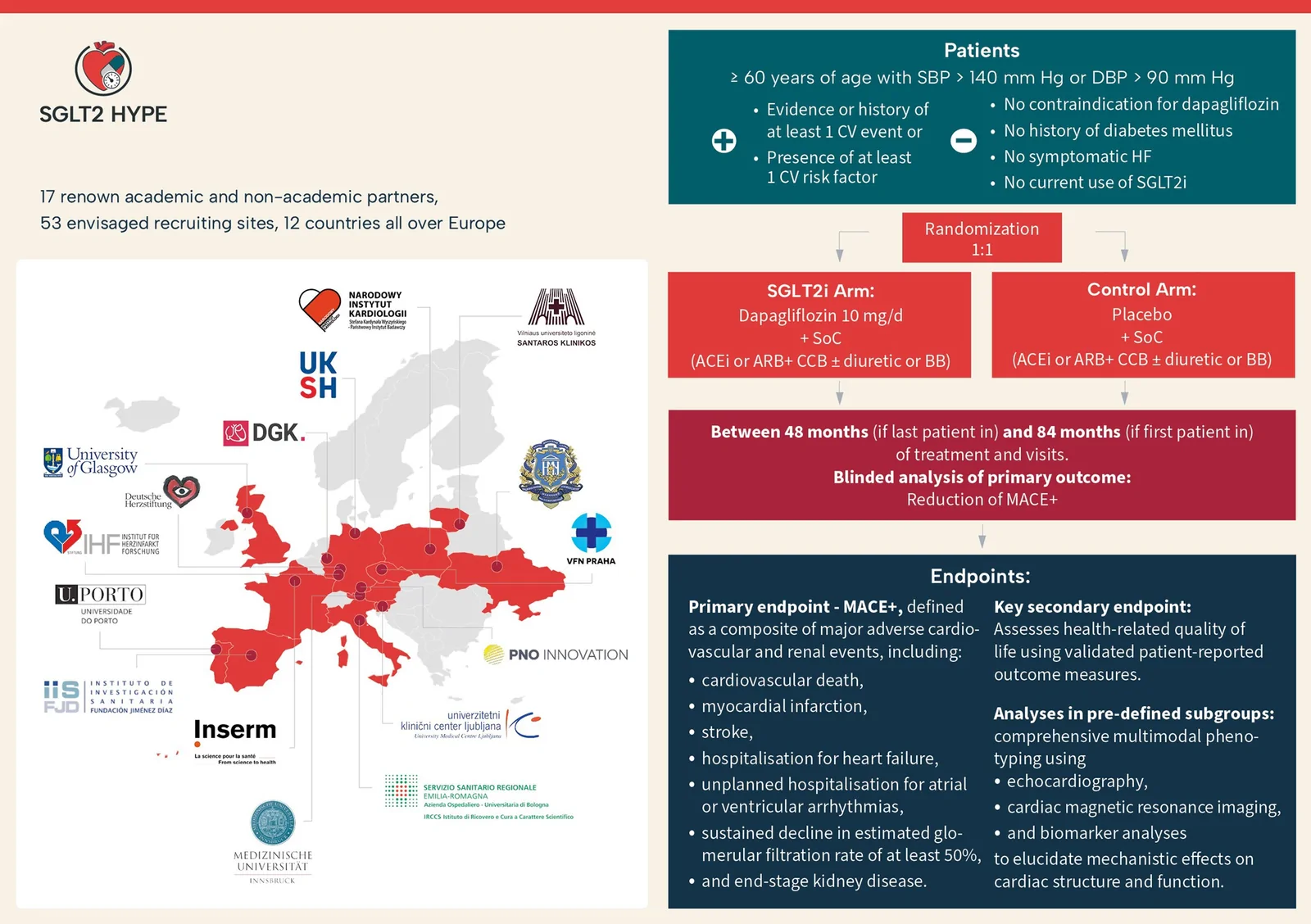
SGLT2 HYPE trial overview

SGLT2 inhibitors have fundamentally changed the treatment of heart failure, chronic kidney disease, and type 2 diabetes. Large cardiovascular outcome trials have consistently demonstrated reductions in cardiovascular mortality, hospitalisation for heart failure, and progression of kidney disease across a wide range of patient populations.^3–6^ Beyond their glucose-lowering effects, SGLT2 inhibitors exert multiple pleiotropic actions that are highly relevant for hypertension (*Figure 1*)^7^:

- sustained reductions in systolic and diastolic blood pressure
- improvement of endothelial function and arterial stiffness
- osmotic diuresis and natriuresis leading to reduced preload and afterload
- favourable effects on cardiac remodelling
- nephroprotection independent of glycaemic status

Meta-analyses have shown blood pressure reductions of approximately 4–6 mmHg with excellent tolerability and additive effects on top of standard antihypertensive therapy.^8–10^ Importantly, these effects are accompanied by consistent reductions in cardiovascular and renal events in other disease settings. Despite this compelling evidence, SGLT2 inhibitors are currently not approved for the treatment of hypertension, largely because no adequately powered cardiovascular outcome trial has specifically addressed this indication. SGLT2 HYPE was designed to close this evidence gap.

## Trial design and methodology

SGLT2 HYPE is a multinational, randomized, double-blind, placebo-controlled, pragmatic outcome trial funded within the EU Horizon programme. The trial will enroll 3162 patients aged ≥60 years with inadequately controlled hypertension and at least one additional cardiovascular risk factor. Participants are recruited from more than 50 centres across 13 European countries, ensuring broad generalisability. Patients are randomized 1:1 to receive Dapagliflozin 10 mg once daily, or a matching placebo, in addition to guideline-directed antihypertensive therapy.

The planned minimum follow-up is four years, allowing robust assessment of long-term outcomes.

The primary endpoint is a composite of major adverse cardiovascular and renal events (MACE+), including cardiovascular death, myocardial infarction, stroke, hospitalisation for heart failure, unplanned hospitalisation for atrial or ventricular arrhythmias, sustained decline in estimated glomerular filtration rate of at least 50%, and end-stage kidney disease. This endpoint reflects the full clinical spectrum of hypertension-related complications and captures outcomes of direct relevance to patients, clinicians, and healthcare systems. A key secondary endpoint assesses health-related quality of life using validated patient-reported outcome measures. In a predefined subgroup, comprehensive multimodal phenotyping using echocardiography, cardiac magnetic resonance imaging, and biomarker analyses will be performed to elucidate mechanistic effects on cardiac structure and function.

## Clinical and societal relevance

If confirmed, SGLT2 HYPE will provide the first outcome-based evidence supporting the use of SGLT2 inhibition in patients with hypertension. The results have the potential to directly inform future European hypertension guidelines and to facilitate evidence-based adoption of SGLT2 inhibition into routine clinical care. Given the high prevalence of hypertension, even moderate relative risk reductions could translate into a substantial absolute reduction in cardiovascular events, disability-adjusted life years, and healthcare utilisation. Modelling data suggests that effective implementation could result in annual healthcare savings in the order of several billion euros across Europe, driven by fewer hospitalisations, reduced progression to advanced cardiovascular and renal diseases, and lower long-term care needs. The trial therefore provides evidence with immediate relevance for both clinical practice and healthcare systems.

## From blood pressure control to outcome-driven care

SGLT2 HYPE represents a pivotal step toward redefining hypertension management. By shifting the therapeutic focus from blood pressure reduction alone to outcome-oriented, disease-modifying treatment, the trial addresses a central limitation of current hypertension care. If successful, SGLT2 HYPE will not only establish a new therapeutic option for patients with hypertension but also provide a framework for future cardiovascular outcome trials in highly prevalent chronic diseases. This approach may mark an important step toward a more effective and sustainable management of hypertension.
